# Optimized Arrangement of Constant Ambient Air Monitoring Stations in the Kanto Region of Japan

**DOI:** 10.3390/ijerph120302950

**Published:** 2015-03-10

**Authors:** Shintaro Shirato, Atsushi Iizuka, Atsushi Mizukoshi, Miyuki Noguchi, Akihiro Yamasaki, Yukio Yanagisawa

**Affiliations:** 1 Department of Environmental Systems, Institute of Frontier Sciences, The University of Tokyo, 5-1-5 Kashiwanoha, Kashiwa, Chiba 277-8563, Japan; E-Mails: hcrmh221@ybb.ne.jp (S.S.); mizukoshi@med.kindai.ac.jp (A.M.); noguchi@ejs.seikei.ac.jp (M.N.); yukio@k.u-tokyo.ac.jp (Y.Y.); 2 Research Center for Sustainable Science and Engineering, Institute of Multidisciplinary Research for Advanced Materials, Tohoku University, 2-1-1 Katahira, Aoba-ku, Sendai, Miyagi 980-8577, Japan; 3 Tokyo Metropolitan Industrial Technology Research Institute, 2-4-10 Aomi, Koto-ku, Tokyo 135-0064, Japan; 4 Department of Environmental Medicine and Behavioral Science, Faculty of Medicine, Kinki University, 377-2, Ohno-higashi, Osakasayama, Osaka, 589-8511, Japan; 5 Department of Materials and Life Science, Faculty of Science and Technology, Seikei University, 3-3-1 Kichijoji-kitamachi, Musashino, Tokyo 180-8633, Japan; E-Mail: akihiro@st.seikei.ac.jp

**Keywords:** constant ambient air monitoring, Kanto region, NO*_x_*, O*_x_*, non-methane hydrocarbon, suspended particulate matter, principle component analysis

## Abstract

Continuous ambient air monitoring systems have been introduced worldwide. However, such monitoring forces autonomous communities to bear a significant financial burden. Thus, it is important to identify pollutant-monitoring stations that are less efficient, while minimizing loss of data quality and mitigating effects on the determination of spatiotemporal trends of pollutants. This study describes a procedure for optimizing a constant ambient air monitoring system in the Kanto region of Japan. Constant ambient air monitoring stations in the area were topologically classified into four groups by cluster analysis and principle component analysis. Then, air pollution characteristics in each area were reviewed using concentration contour maps and average pollution concentrations. We then introduced three simple criteria to reduce the number of monitoring stations: (1) retain the monitoring station if there were similarities between its data and average data of the group to which it belongs; (2) retain the station if its data showed higher concentrations; and (3) retain the station if the monitored concentration levels had an increasing trend. With this procedure, the total number of air monitoring stations in suburban and urban areas was reduced by 36.5%. The introduction of three new types of monitoring stations is proposed, namely, mobile, for local non-methane hydrocarbon pollution, and O*_x_*-prioritized.

## 1. Introduction

Continuous ambient air monitoring systems have been introduced worldwide [1,2,3,4,5]. Data obtained from such monitoring can provide important information about surrounding atmospheric pollution. For example, Li *et al.* [6] developed a simple method to assess the rate of O_3_ exceeding standards the following day with statistical analysis of long-term data at a site in an air quality monitoring network. Lu *et al.* [7] proposed a revised air quality index to provide information on current air quality from monitoring network data. However, this monitoring forces autonomous communities to bear a significant financial burden. Thus, it is important to identify pollutant-monitoring stations that are less efficient while minimizing loss of data quality and mitigating effects on determination of spatiotemporal trends of pollutants. There have been trials to reexamine the efficacy of existing constant ambient air monitoring stations based on operating standards of the Ministry of the Environment (Japan) [8], such as in the cities of Shizuoka and Funabashi, and in Hiroshima Prefecture of Japan [9,10,11]. However, there are no current reliable guidelines regarding the optimal method by which this can be achieved.

In our previous study [12], we applied cluster analysis to continuous ambient air monitoring data in 1996 and 2006 in the Kanto region of Japan, based on the expectation that similarities in site characteristics and pollutant behaviors could be identified, and that monitoring stations could be grouped topologically. As expected, cluster analysis confirmed that ambient monitoring stations could be clustered topologically for NO*x* and O*x*. When ambient air monitoring stations can be reasonably grouped, then a method for reducing both the number of monitoring stations and pollutants monitored should be possible.

In the present study, we considered further grouping of the stations in Kanto using principal component analysis (PCA). PCA provides the most significant variables relating to the source of the variation. PCA is used in many studies related to continuous ambient air monitoring data [13,14,15,16]. Regarding monitoring system optimization, Pires *et al.* [17,18] applied PCA and cluster analysis to SO_2_, PM_10_, CO, NO_2_ and O_3_ at 10–12 monitoring sites within an air quality monitoring network on an annual basis, to identify city areas with similar pollution behaviors and locate emission sources for management of air quality monitoring systems. Pires *et al.* [19] also applied PCA to data divided into quarter years to consider annual variation of air pollutant behaviors and identified redundant air quality measurements. Ibarra-Berastegi *et al.* [20] developed a method to identify redundant sensors and evaluate network capability to correctly monitor and represent SO_2_ fields in Bilbao, in the framework of a continuous network optimization process using three techniques, Self-Organizing Maps (SOMs), cluster analysis, and PCA. Among those, information obtained via PCA can be helpful not only for the purpose in that study but also to throw light on major mechanisms involved. Lu *et al.* [21] applied PCA and cluster analysis on three pollutants (SO_2_, RSP, NO_2_) at fourteen stations for management of air quality monitoring network.

In the present study, the Kanto region was divided into four areas by PCA, and air pollution characteristics in each area were addressed. We then introduced three simple criteria: (1) retain the monitoring station if there were similarities between its data and average data of the area to which it belongs; (2) retain the station if its data showed higher concentrations; and (3) retain the station if the monitored concentration levels had an increasing trend, to reduce the number of monitoring stations. Over 30% of the stations were successfully removed by adopting the above criteria.

## 2. Method

### 2.1. Air Monitoring Data

**Figure 1:**
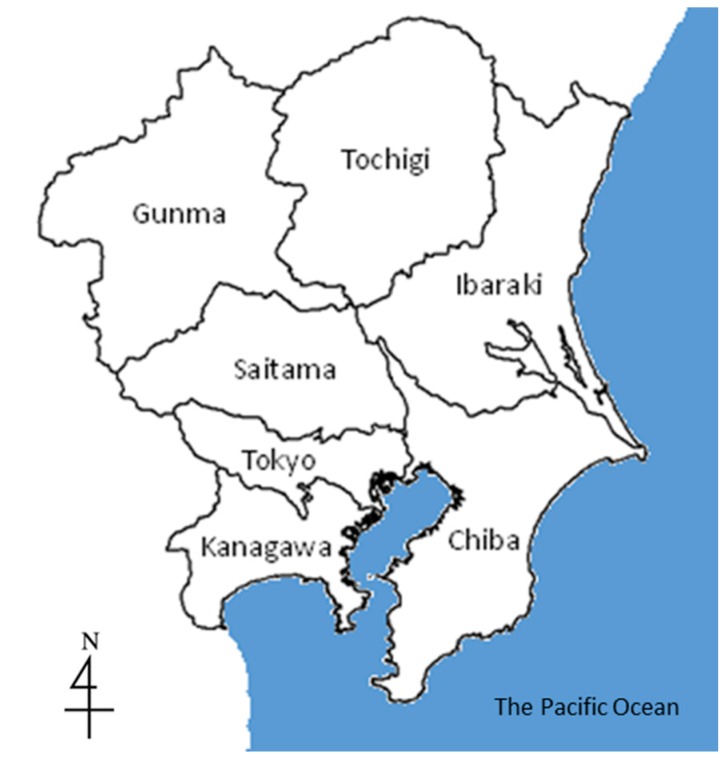
Map of seven prefectures of Kanto region in Japan.

The air monitoring dataset was the same as that used in our previous study [12]. That study also focused on the Kanto region and included the seven prefectures of Tokyo, Gunma, Tochigi, Ibaraki, Chiba, Saitama, and Kanagawa (Figure 1). We used continuous ambient air monitoring data from 476 stations during fiscal years 1996 and 2006. The two years were selected to evaluate the influence of the Automobile NO*_x_*/PM Act of Japan and diesel control, in which a series of raw revisions were made from 1999 to 2005. There are two types of ambient air monitoring stations in Japan, general environmental and vehicle emission [22]. The latter are distributed near major roadways to monitor air pollution from vehicle emissions. Data from both types of stations were used.

### 2.2. Grouping of Air Monitoring Stations

In our previous study [12], we used cluster analysis and succeeded in topologically grouping air monitoring stations in the Kanto region. Detailed information on the cluster analysis of that study has been described elsewhere [12]. In the present study, we pursue further topological grouping of the air monitoring stations in the region using principal component analysis (PCA), because some of the eight clusters still showed pollution trends similar to other clusters. For this grouping, as a base, we chose eight-group clustering by NO*_x_* general environmental air monitoring stations. Because this clustering according to pollutant was topologically very clear, the number of stations at which NO*_x_* was monitored was sufficiently large (356 of 476 stations). However, the selection of which pollutant to use for station grouping was arbitrary. In the grouping procedure, a station at which NO*_x_* was not monitored was classified in the same group as the nearest NO*_x_* monitoring station.

### 2.3. Principal Component Analysis *(*PCA*)*

SPSS Statistics 17.0 (SPSS Inc., Chicago, IL, USA) was used for the PCA. Data matrices were prepared from average pollutant concentrations of each cluster. In each matrix, the element in the *i*th row and *j*th column represents average concentration of the *i*th pollutant (NO*_x_*, suspended particulate matter (SPM), non-methane hydrocarbon (NMHC), and others) in the *j*th cluster.

### 2.4. Characterization of Air Pollution in Each Area

After division by PCA, air pollution characteristics of each area were reviewed using concentration contour maps and average pollution concentrations in the area. The maps are for pollutant concentrations in which the monitoring month is on the abscissa and time on the ordinate. Several studies have used this method for air pollution analysis [23,24]. Using these maps, seasonal and diurnal variations of pollutant concentrations can be examined visually.

### 2.5. Reexamination of Constant Air Monitoring Stations

By PCA of the average pollution concentrations, air monitoring stations in the Kanto region were classified into four groups: rural, semi-rural, suburban, and urban areas. We first examined the density of stations in each area. We then chose the suburban and urban areas for station reduction. To reexamine the stations, we adapted the following three simple criteria:

(1) Retain the station if there were similarities between its monitored data and average data of the group to which it belonged;
(2) Retain the station if the monitored data had high concentrations;
(3) Retain the station if the concentration levels had an increasing trend.

For the first criterion, Euclidean distances between each element of monitored and average data in the topological group matrix were calculated. Only the top 5%–15% of stations with smallest Euclidean distances were retained (defined as average station). For the second criterion, the top 5%–15% of stations with highest annual average concentrations in 1996 and 2006 were retained. For the third criterion, the top 5%–15% of stations with the highest ratio of annual average concentrations in the same period were retained. The retention ratio for each criterion varied within 5%–15%. These examinations were conducted for all pollutants and if any criterion was applied to a station, it was retained and monitoring for all pollutants was assumed continuous. Based on the results and their evaluation, we propose the introduction of new types of monitoring stations for the area by considering local air pollution.

## 3. Results and Discussion

### 3.1. Grouping by Cluster Analysis and Subsequent Principal Component Analysis

**Figure 2:**
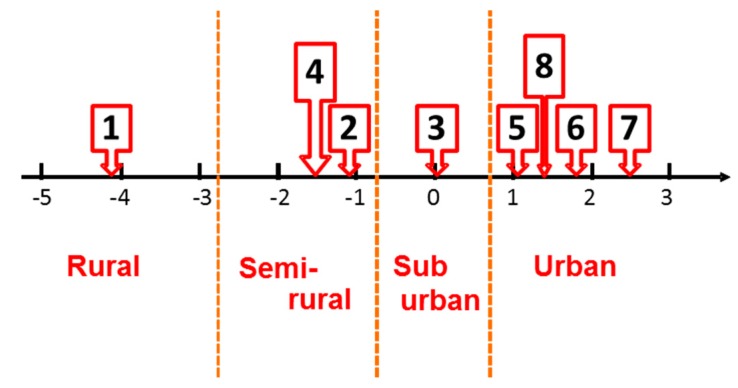
Plot of first principal component score of eight clusters and re-grouping into four groups.

**Figure 3:**
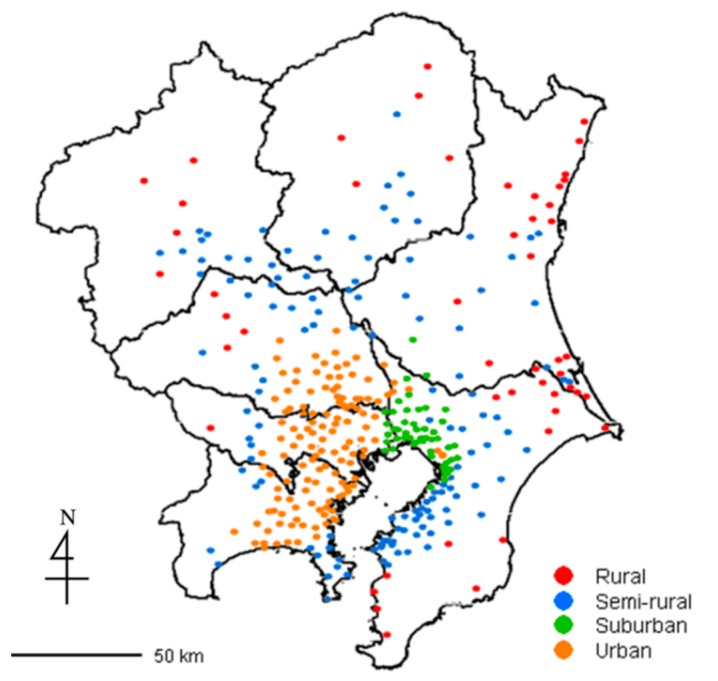
Geographic mapping of four groups of constant ambient air monitoring stations.

The contribution ratio of the first principal component was 93.4% when treating the eight clusters divided according to NO*_x_* monitoring data in 2006, which were monitored by general environmental air monitoring stations. This means that the pollution trend of all data can mostly be explained by only the first principle component. Figure 2 shows a plot of the first principal component score for the eight clusters. We interpreted the axis as the degree of urbanization in the Kanto region and re-grouped the eight clusters into four groups: rural, semi-rural, suburban, and urban areas. Figure 3 illustrates the geographically classified mapping of the four groups of ambient air monitoring stations. The grouping appears very reasonable when considering the actual degree of urbanization in the area.

### 3.2. Air Pollution Characteristics in Each Area

#### 3.2.1. Rural Area

**Figure 4:**
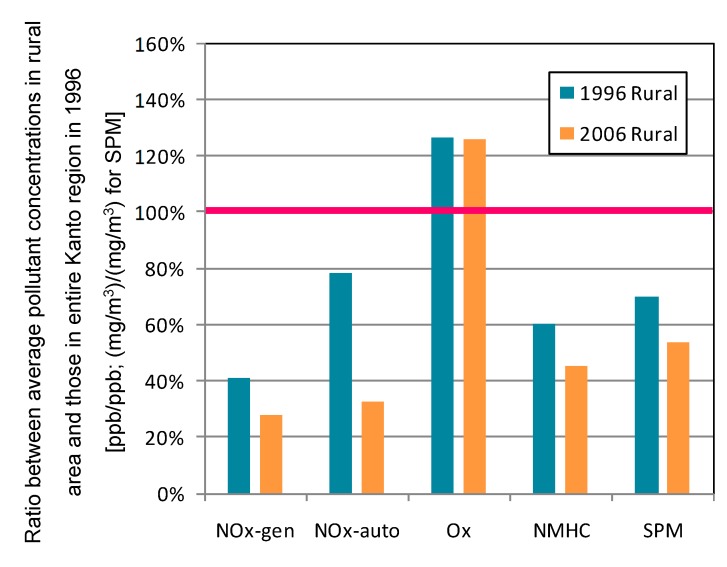
Ratio between average pollutant concentrations in rural area and those in the entire Kanto region in 1996. Average concentration of entire Kanto region in 1996 corresponds to 100%.

The rural area corresponds to the first cluster divided by NO*_x_* monitoring data in 2006 from the general environmental air monitoring stations (for the clustering approach, see [12]). This area is the most remote in the Kanto region. Figure 4 shows the ratio between average pollutant concentrations in the rural area and those in the entire Kanto region in 1996. Except for O*_x_*, pollutant concentrations are lower than the entire Kanto region average, and had a decreasing trend over the decade. NO*_x_* concentration monitored by the vehicle emission monitoring stations especially decreased. This is attributable to higher NO*_x_* concentrations (>120 ppb) monitored by two stations in the area during 1996. The average NO*_x_* concentration at these two stations dramatically decreased to less than 40 ppb in 2006. The ratio of decrease in SPM concentration in the rural area was smaller than that of the entire region (100%→64.3%). This is explained by the difference in contribution ratio of SPM emitted by automobiles. In the urban area, SPM concentration decreased greatly because of the revised Automobile NO*_x_*/PM Act of Japan and diesel control. Thus, the smaller contribution ratio of SPM emitted by automobiles in the rural area resulted in the smaller ratio of decrease in SPM concentration.

**Figure 5:**
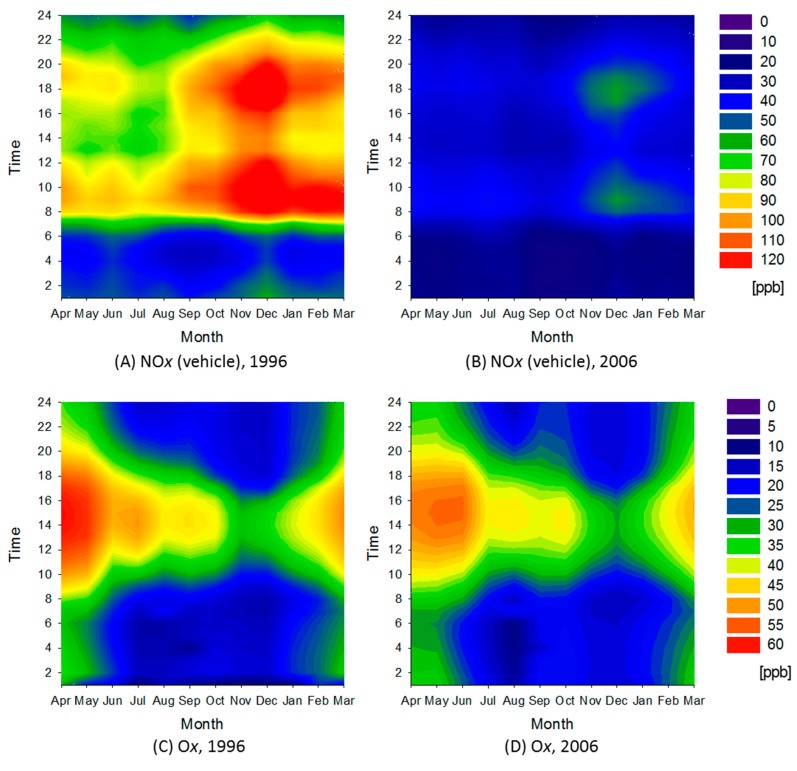
Contour maps for NO*_x_* monitoring data from vehicle emission monitoring stations: (**A**) in 1996 and (**B**) in 2006; and for O*_x_* (**C**) in 1996 and (**D**) in 2006.

Figure 5 shows contour maps of NO*_x_* monitoring data from the vehicle emission monitoring stations and O*_x_* monitoring data in the rural area. The NO*_x_* maps for the rural area are very different from those in other areas. Throughout the year, NO*_x_* concentrations observed before 06:00 A.M. were very low, and rapidly increased between 07:00 A.M. and 08:00 A.M. One of the reasons for this is the time lag for traffic peaks in the rural area, which is far from the urban area with its greater traffic. Contour maps for NO*_x_* monitoring data from the general environmental air monitoring stations showed no significant characteristics (data not shown). O*_x_* peak concentration during spring and summer daytime decreased over the decade. Table 1 summarizes the average total number of times in which O*_x_* concentrations greater than 60 and 120 ppb were observed in each area. The frequency for concentrations greater than 120 ppb decreased during the decade only in the rural area. We anticipate that meteorological and geographic factors involved in this finding will be investigated.

#### 3.2.2. Semi-Rural Area

**Figure 6:**
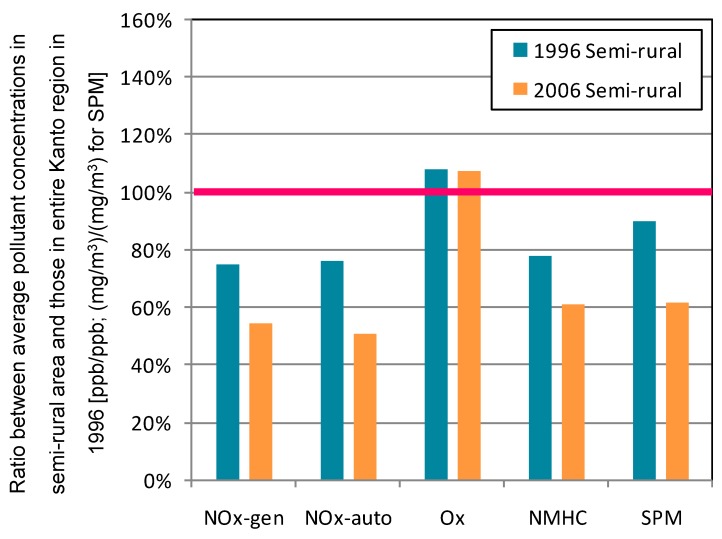
Ratio between average pollutant concentrations in semi-rural area and those in entire Kanto region during 1996. Average concentration in entire Kanto region during 1996 corresponds to 100%.

The semi-rural area corresponding to clusters 2 and 4 divided by 2006 NO*_x_* data from the general environmental air monitoring stations. This area surrounds the urban area in the Kanto region. Figure 6 shows the ratio between average pollutant concentrations in the semi-rural area and those in the entire Kanto region during 1996.

**Table 1:** Average total number of times in which O*_x_* concentrations greater than 60 and 120 ppb were observed in each area.

Area	Cluster	Average Total Number of Times with Observed O*x* Concentrations Greater than 60 ppb			Average Total Number of Times with Observed O*x* Concentrations Greater than 120 ppb		
		1996		2006	1996		2006
Rural	NO*x*-gen1	485	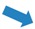	374	7.6	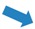	4.4
Semi-rural	NO*x*-gen2	400	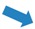	374	7.3	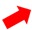	10
	NO*x*-gen4	369	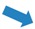	319	4.0	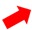	7.0
Suburban	NO*x*-gen3	174	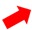	267	1.3	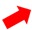	9.5
Urban	NO*x*-gen5	238	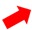	474	4.8	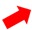	24
	NO*x*-gen6	90	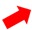	314	0.2	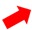	9.6
	NO*x*-gen7	138	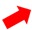	277	1.8	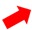	11
	NO*x*-gen8	322	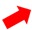	450	2.6	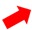	17

In the semi-rural area, average O*_x_* concentration did not change. Except for O*_x_*, pollutant concentrations were lower than the entire region average and had a decreasing trend over the decade. However, ratios of decrease of concentrations in the semi-rural area were smaller than those of the entire region. Counteracting effects of the pollutants were relatively weak in this area.

**Figure 7:**
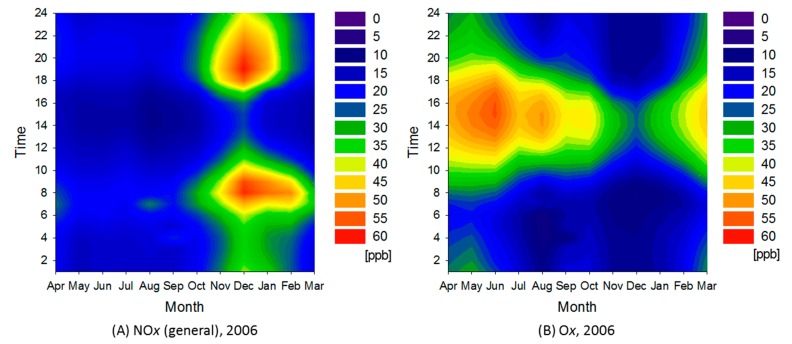
Contour maps for NO*_x_* and O*_x_* in cluster 2 for 2006.

Figure 7 shows contour maps for NO*_x_* and O*_x_* in cluster 2 for 2006. In the semi-rural area, average NO*_x_* concentration was about twice that in the rural area. Greater pollution in winter is clearly seen in the map. If we compare the two contour maps, it is obvious that lower NO*_x_* concentrations were contemporaneous with higher O*_x_* concentrations. This trend was observed in all areas of the region. Table 1 shows that the frequency of O*_x_* concentration in excess of 120 ppb increased, whereas that greater than 60 ppb decreased.

#### 3.2.3. Suburban Area

**Figure 8:**
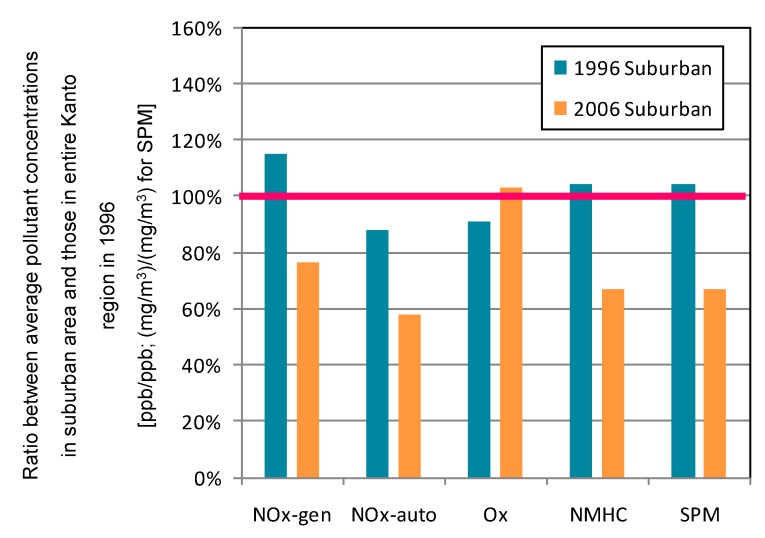
Ratio between average pollutant concentrations in suburban area and those in entire Kanto region during 1996. Average concentration of entire Kanto region in 1996 corresponds to 100%.

**Figure 9:**
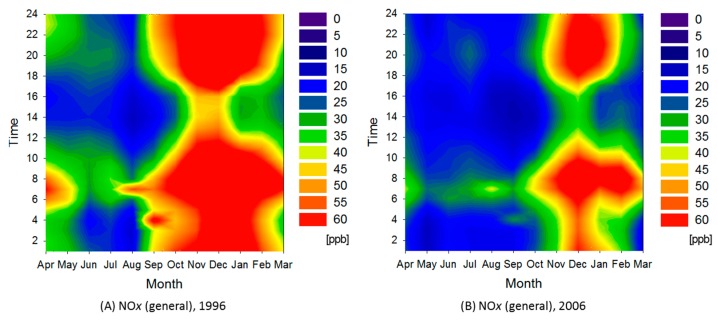

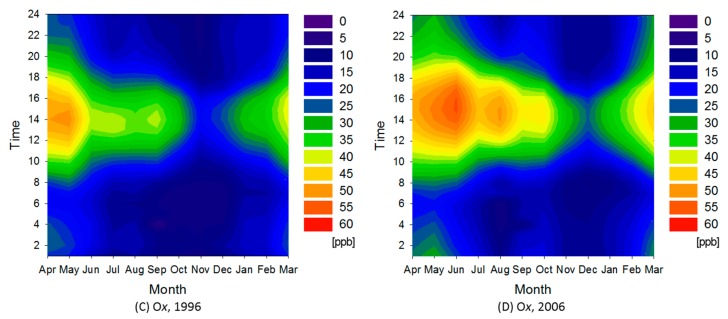
Contour maps for NO*_x_* and O*_x_* in 1996 and 2006.

The suburban area corresponds to cluster 3. This area is in the northwest part of Chiba Prefecture. Figure 8 shows the ratio between average pollutant concentrations in the suburban area and those in the entire Kanto region during 1996. In the suburban area, average O*_x_* concentration increased about 13%. Other pollutant concentrations declined about 35%. The ratio of decrease for NO*_x_* was notably higher than the average for the entire Kanto area. There was a trend of higher NO*_x_* ratios in urbanized areas relative to the rural area. Figure 9 shows contour maps for NO*_x_* and O*_x_* in the suburban area for 1996 and 2006. Average NO*_x_* concentration in that area was intermediate to those of the semi-rural and urban areas. It is seen that overall NO*_x_* concentrations in the suburban area decreased over the decade, whereas those of O*_x_* increased overall. Observed NMHC concentrations dramatically decreased over the period, reaching the same level observed in the semi-rural area during 2006. The temporal trend was also the same as that of the semi-rural area. Within the decade, the SPM concentration maximum in the suburban area transitioned from winter to summer; the same trend was observed in the suburban area (data not shown).

#### 3.2.4. Urban Area

**Figure 10:**
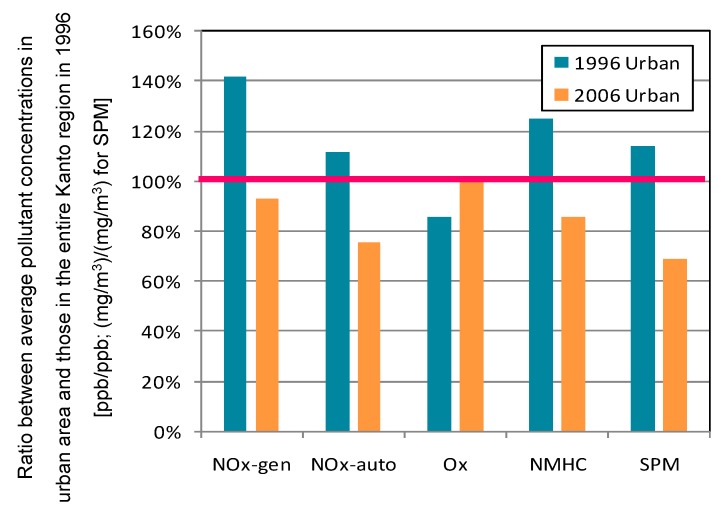
Ratio between average pollutant concentrations in urban area and those in entire Kanto region during 1996. Average concentration of entire Kanto region in 1996 corresponds to 100%.

**Figure 11:**
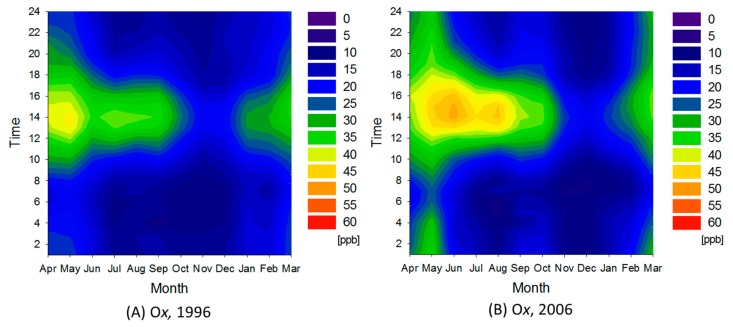
Contour maps for O*_x_* in cluster 7 during 1996 and 2006.

The urban area consisted of clusters 5 through 8, and extended from Saitama to Kanagawa prefectures. Figure 10 shows the ratio between average pollutant concentrations in the urban area and those in the entire Kanto region during 1996. In the urban area, average O*_x_* concentration increased about 16% and the frequency of O*_x_* concentrations higher than the environmental standard (60 ppb) dramatically increased over the decade (Table 1). Other pollutant concentrations declined about 30%–40%. The ratio of decrease for SPM was notably higher than the average for the entire Kanto region. The urban area covers almost all that targeted by the revised Automobile NO*_x_*/PM Act of Japan and diesel control, so the decreasing trend is attributable to these regulations. Figure 11 shows contour maps for O*_x_* in the urban area in 1996 and 2006. O*_x_* concentration increased particularly in spring through summer. As a result, average O*_x_* concentration in the urban area approached the average of the entire Kanto region.

#### 3.2.5. Summary of Air Pollution Characteristics in Each Area

From the PCA of average pollution concentrations, air monitoring stations in the Kanto region were classified into four groups: rural, semi-rural, suburban, and urban areas. Air pollution characteristics in each area were reviewed using average pollution concentrations and concentration contour maps.

• In the rural area, a smaller ratio of decrease in SPM concentrations was attributable to the smaller contribution of automobiles to pollutant generation.
• In the rural area, average O*_x_* concentration did not decrease; however, the frequency of O*_x_* at higher concentration diminished.
• In the semi-rural area except for O*_x_*, the ratio of decrease in pollutant concentrations was smaller than those in the entire Kanto region. In that area, effects counteracting these pollutants were weak.
• In the suburban area, O*_x_* pollution worsened while NMHC pollution dramatically improved. NO*_x_* and SPM pollution also improved.
• In the urban area, SPM pollution was much improved compared with other areas.
• In the urban area, the frequency of O*_x_* concentrations higher than the environmental standard (60 ppb) dramatically increased over the decade.

### 3.3. Reexamination of Constant Air Monitoring Stations

Air monitoring stations in the Kanto region were similarly classified into four groups via the PCA of average pollution concentrations (rural, semi-rural, suburban, and urban areas). We first examined the density of stations in each area (Table 2). As seen in the table, densities in the suburban and urban areas were very high, so we anticipate removing some stations. We therefore decided to choose the suburban and urban areas for station reduction. These two areas cover the southeast part of Saitama Prefecture, Tokyo (east of Hachioji), northwestern Chiba Prefecture, and Kanagawa Prefecture (except its western part and the Miura Peninsula). There were 293 air monitoring stations in the two areas. Station numbers in 2006 for each pollutant were as follows: NO*_x_* (general) 180, NO*_x_* (vehicle) 109, O*_x_* 160, NMHC 113, and SPM 289.

**Table 2:** Density of monitoring stations in each area.

Area	Density of Air Monitoring Stations (number/1000 km^2^)
Rural	3.8
Semi-rural	12
Suburban	64
Urban	56

**Figure 12:**
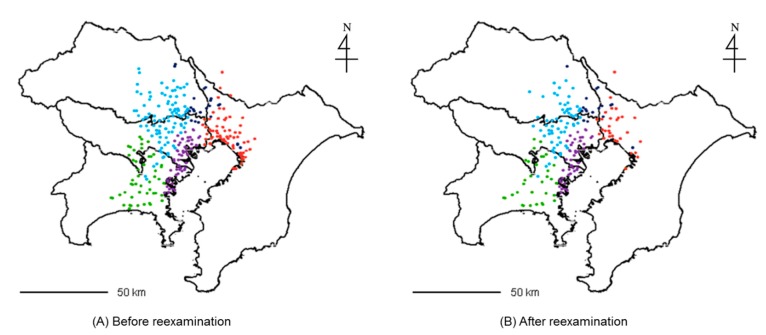
Mapping of air monitoring stations in semi-urban and urban areas before and after reexamination (retention ratios for the criteria were all 10%).

Then, for the two areas, we adapted the retention criteria to reduce the number of stations. Table 3 shows the number of stations in the semi-urban and urban areas before and after reexamination. Retention ratios for the three criteria were all set to 10%. The total air monitoring station number was reduced 36.5% by the reexamination, and the number of stations at which each pollutant was monitored also declined 30%–40%. Figure 12 maps the stations in the semi-urban and urban areas before and after reexamination. Table 4 shows the average concentration of each pollutant before and after the reexamination. Slight decreases in average NO*x* concentrations were observed. However, the ratio of decrease of NO*x* monitored at general environmental air monitoring stations was less than 8%.

No significant change was observed for other pollutants. Table 5 shows the number of stations for various retention ratios of the criteria. Changing the retention ratio altered the number of stations retained.

**Table 3:** Number of air monitoring stations in semi-urban and urban areas before and after reexamination (retention ratios for criteria were all 10%).

Station Type and Monitored Pollutant	Before Reexamination	After Reexamination	Ratio of Decrease (%)
Total air monitoring stations	293	186	36.5
Vehicle emission monitoring stations	109	65	40.4
NO*x* (general)	180	120	33.3
NO*x* (vehicle)	109	65	40.4
O*x*	160	111	30.6
NMHC	113	79	30.1
SPM	289	185	36.0

**Table 4:** Average concentration of each pollutant before and after reexamination.

Monitored Pollutant	Average Concentration (ppb; mg/m^3^ for SPM)	
	Before Reexamination	After Reexamination
NO*x* (general)	34.8	32.3
NO*x* (vehicle)	69.9	61.7
O*x*	23.7	23.6
NMHC	268.5	267.3
SPM	31.1	31.0

### 3.4. Recommendations for More Efficient Continuous Air Monitoring System in Kanto Region

Based on the foregoing results and discussion, we suggest introducing new types of monitoring stations in the area, considering local air pollution. In general, the continuous ambient air monitoring stations were installed to ascertain the achievement of environmental standards or to recognize the effects of countermeasures for air pollution. Therefore, these stations were usually located to discern the general pollution trend in the area. However, it is also important to determine local high-level air pollution from the standpoint of its adverse effects on human and atmospheric chemistry. Therefore, we suggest new types of stations.

**Table 5:** Number of air monitoring stations for various retention ratios of the criteria.

Retention Ratio of the Criteria (%)	Retained	Removed	Ratio of Decrease (%)
5	121	172	58.7
10	186	107	36.5
15	233	60	20.5

#### 3.4.1. Mobile Monitoring Stations

The first new type of station is mobile monitoring. The concept underlying such stations has been introduced in the literature [22]. Large numbers of stations may be required to investigate local air pollution. To this end, mobile stations would be effective. These stations are prefabricated, with analytical equipment that can be easily transported. With such stations, we can save on costs of new air monitoring station construction. The analytical equipment can come from that of removed stations. The mobile stations can be also used for determination of locations for installation of new fixed stations.

#### 3.4.2. Monitoring Stations for Local NMHC Pollution

During analysis of the local characteristics of NMHC pollution in the Kanto region, we identified monitoring stations with higher NMHC concentrations, even though they were far from major traffic arteries. Investigation of the cause of such high concentrations would have great significance, so we propose another new type of monitoring station for this purpose. For example, the stations with the top ten average NMHC concentrations could be selected as those for monitoring local NMHC pollution. Detailed analysis of volatile organic compounds (VOCs; including carbonyl and aromatic compounds) could be conducted for the stations. In such a way, we could determine the reason for the elevated NMHC concentrations and quickly address strong NMHC emission sources around the stations.

#### 3.4.3. O*_x_*-Prioritized Monitoring Stations

The O*_x_* air pollution situation is changing, and related countermeasures are difficult to implement. To learn more about the atmospheric chemistry of O*_x_* generation, we propose the introduction of O*_x_*-prioritized monitoring stations. For example, stations at which higher O*_x_* concentrations have been continuously measured could be selected as O*_x_*-prioritized ones. Then, detailed analysis of VOCs, NO*_x_* monitoring, and weather data would also be conducted for the stations. If we set the criteria for the selection as “*over 20 h observation with greater than 120 ppb O_x_ in 2006 and observed frequency increased over that in 1996*”, 58 monitoring stations would be selected as the O*_x_*-prioritized monitoring stations (31 stations using more than 30 h and nine stations using more than 40 hours).

## 4. Conclusions

Ambient air monitoring stations in the Kanto region were divided into four topological groups, using cluster analysis and subsequent PCA. Then, air pollution characteristics in each divided area were reviewed using concentration contour maps and average pollution concentrations there. Afterward, we introduced three simple criteria. Using this procedure, total station number in the suburban and urban areas was decreased by 36.5%. The introduction of three new types of monitoring stations was proposed, *i.e.*, mobile, local NMHC pollution, and O*_x_*-prioritized.
